# Autophagy Promotes the Survival of Adipose Mesenchymal Stem/Stromal Cells and Enhances Their Therapeutic Effects in Cisplatin-Induced Liver Injury via Modulating TGF-β1/Smad and PI3K/AKT Signaling Pathways

**DOI:** 10.3390/cells10092475

**Published:** 2021-09-18

**Authors:** Eman Mohamad El Nashar, Mansour Abdullah Alghamdi, Wardah Abdullah Alasmari, Mohamed M. A. Hussein, Eman Hamza, Reham Ismail Taha, Mona M. Ahmed, Khulood Mohammed Al-Khater, Ahmed Abdelfattah-Hassan

**Affiliations:** 1 Department of Anatomy, College of Medicine, King Khalid University, Abha 61421, Saudi Arabia; m.alghamdi@kku.edu.sa; 2 Department of Histology and Cell Biology, Faculty of Medicine, Benha University, Benha 13511, Egypt; 3 Genomics and Personalized Medicine Unit, College of Medicine, King Khalid University, Abha 61421, Saudi Arabia; 4 Department of Anatomy, Faculty of Medicine, Umm Al-Qura University, Makkah 24230, Saudi Arabia; waasmari@uqu.edu.sa; 5 Biochemistry Department, Faculty of Veterinary Medicine, Zagazig University, Zagazig 44519, Egypt; hamza_vet@yahoo.com; 6 Department of Medical Biochemistry, Faculty of Medicine, Mansoura University, Mansoura 35516, Egypt; emanhamza2010@yahoo.com or; 7 Department of Biochemistry and Molecular Biology, Horus University, Damietta 34511, Egypt; 8 Department of Anatomy, Faculty of Medicine, Mansoura University, Mansoura 35516, Egypt; anamed1076@yahoo.com; 9 Forensic Medicine and Toxicology Department, Faculty of Veterinary Medicine, Zagazig University, Zagazig 44519, Egypt; drmonaforensic@yahoo.com; 10 Department of Anatomy, College of Medicine, Imam Abdulrahman Bin Faisal University, P.O. Box 1982, Dammam 31441, Saudi Arabia; kalkhater@iau.edu.sa; 11 Department of Anatomy and Embryology, Faculty of Veterinary Medicine, Zagazig University, Zagazig 44519, Egypt; 12 Biomedical Sciences Program, Zewail City of Science and Technology, University of Science and Technology, Giza 12578, Egypt

**Keywords:** ADSCs, cell-based therapy, rapamycin, apoptosis, liver damage, hepatoprotective pathways

## Abstract

Autophagy is a key metabolic process where cells can recycle its proteins and organelles to regenerate its own cellular building blocks. Chemotherapy is indispensable for cancer treatment but associated with various side-effects, including organ damage. Stem cell-based therapy is a promising approach for reducing chemotherapeutic side effects, however, one of its main culprits is the poor survival of transplanted stem cells in damaged tissues. Here, we aimed to test the effects of activating autophagy in adipose-derived mesenchymal stem/stromal cells (ADSCs) on the survival of ADSCs, and their therapeutic value in cisplatin-induced liver injury model. Autophagy was activated in ADSCs by rapamycin (50 nM/L) for two hours before transplantation and were compared to non-preconditioned ADSCs. Rapamycin preconditioning resulted in activated autophagy and improved survival of ADSCs achieved by increased autophagosomes, upregulated autophagy-specific LC3-II gene, decreased protein degradation/ubiquitination by downregulated p62 gene, downregulated mTOR gene, and finally, upregulated antiapoptotic BCL-2 gene. In addition, autophagic ADSCs transplantation in the cisplatin liver injury model, liver biochemical parameters (AST, ALT and albumin), lipid peroxidation (MDA), antioxidant profile (SOD and GPX) and histopathological picture were improved, approaching near-normal conditions. These promising autophagic ADSCs effects were achieved by modulation of components in TGF-β1/Smad and PI3K-AKT signaling pathways, besides reducing NF-κB gene expression (marker for inflammation), reducing TGF-β1 levels (marker for fibrosis) and increasing SDF-1 levels (liver regeneration marker) in liver. Therefore, current results highlight the importance of autophagy in augmenting the therapeutic potential of stem cell therapy in alleviating cisplatin-associated liver damage and opens the path for improved cell-based therapies, in general, and with chemotherapeutics, in particular.

## 1. Introduction

Over the last 30 years, the survival rates of cancer have increased from 49% to 68%, with recent advancements in early detection and therapy of cancer [1]. Although numbers of cancer survivors are increasing worldwide, the side effects of chemotherapy sometimes supersede the effects of the chemotherapeutics [2]. The most prominent side effects include multi-organ injury/toxicity, which occurs in liver [3], kidney [4], CNS [5], and other vital organs. Although the exact mechanism of cytotoxicity of chemotherapeutic agents, including cisplatin, is not fully understood, one of the main causes is the generation of different reactive oxygen species (ROS), including superoxide anion (O_*_^−^), hydrogen peroxide (H_2_O_2_) and the hydroxyl radical (OH^−^) [6], which interact with DNA, lipids and proteins inside the cells, resulting in oxidative stress. The latter is manifested by DNA damage, lipid peroxidation, protein degradation and leakage of ions in the affected body cells [7]. Cisplatin-induced toxicity also leads to apoptosis and cell death [8], and lastly, it exaggerates local tissue inflammation by stimulation of proinflammatory cytokines production [9]. Cisplatin-induced hepatotoxicity has been associated with a low rate of serum enzyme elevations during therapy [10]. In addition, hepatocellular liver injury, steatosis and necrosis (steatohepatitis) was found by liver biopsy in patients who started a regimen of cisplatin [11]. Previous studies demonstrated that oxidative stress is one of the main mechanisms of hepatotoxicity in patients receiving chemotherapy [12]. Various treatment modalities have been proposed to overcome cytotoxicity secondary to chemotherapeutics. Stem cell therapy is one of the most prominent candidates. However, to date the effect of stem cell transplantation to repair cisplatin-induced liver injury/toxicity remains debatable with a lack of studies addressing this subject.

Stem cells (SCs) are widely accepted as promising therapeutic tools for treating various autoimmune, ischemic and degenerative diseases due to their inherent differentiation capacity, immunomodulation, and pro-angiogenic characteristics. Mesenchymal stem cells (MSCs) can be easily obtained from bone marrow (BM-MSCs) or adipose tissue (ADSCs) and possess self-renewal and differentiation potential [13]. In addition, MSCs have shown several additional therapeutic advantages such as their ability to migrate to injured tissues, various secreted immunosuppressive factors, and safety with no rejection after infusion of allogeneic MSCs [14]. Several reports demonstrated the beneficial effects of naïve MSCs on the attenuation of platinum-induced injuries. However, one of the chief hurdles for successful MSCs transplantation is the poor survival rate after MSCs settle within the inflamed and hypoxic environments of damaged tissues [15]. As it was previously noted, transplanted MSCs underwent apoptosis post-transplantation in vivo [16]. Nevertheless, recent studies provided evidence that autophagy activation in MSCs directly before their transplantation enhanced their viability, post-transplantation survival, promoted their differentiation, immunomodulation, and pro-angiogenic capabilities, and henceforth, improved the overall tissue repair potential [17].

Autophagy is an important intracellular process in which cellular components such as proteins, lipids and organelles are recycled by means of autophagosomes [18]. Thus, instead of being degraded inside the lysosomes, these recycled products inside autophagosomes are returned to the cytoplasm to be re-used for maintenance of cellular homeostasis. In addition, autophagy represents a basic metabolic process that responds to deprivation from nutrients and/or oxygen and acts as an intracellular quality control mechanism [19]. Recently, numerous reports demonstrated the crucial role of autophagy in various cell types within the body and in human disease and aging [20]. Moreover, autophagy emerged as a key process necessary for maintenance and proper functioning of several stem cell types via promoting their quiescence, maintaining their stemness and self-renewal and mediating their differentiation [21,22]. Autophagy also was also found to protect stem cells from senescence by restoring their regenerative capacity and reducing their mitochondrial dysfunction and oxidative stress [23]. In addition, it was demonstrated that autophagy can be induced in vitro in MSCs by using rapamycin prior to their transplantation, and the results showed that there was a significant increase of autophagy-related gene expression [24]. In a recent study, using a rat model of ischemia-reperfusion myocardial injury, the incubation of BM-MSCs with rapamycin activated their endogenous autophagic processes, protected the cells from apoptosis and enhanced their survival and differentiation following transplantation into the ischemic myocardial areas [25]. 

Until now, few data exist in the literature on the modulation of autophagy as a new strategy for achieving better MSCs transplantation and enhancing the functional/therapeutic characteristics of transplanted MSCs. Attempts to clarify the cytoprotective effects of activated-autophagy were made in few animal models (such as, diabetes, myocardial infarction and graft-versus-host disease), but no data exists in the literature on the role of autophagy in enhancing ADSCs therapeutic use in liver injury induced by cisplatin, and thus, more research is still needed. Therefore, the aim of the present study was to investigate the role of activated autophagy in improving the survival and therapeutic potential of ADSCs after transplantation in a rat model with chemotherapy (cisplatin)-induced liver injury.

## 2. Materials and Methods

### 2.1. Study Animals

This study included fifty-five Sprague–Dawley female rats (8–10 weeks of age and weighted 180 ± 20 g) were used in this study, the rats were obtained from the Experimental Animal Unit, Faculty of Veterinary Medicine, Benha University, Egypt. Study rats were reared in a controlled environment, maintained under a light/dark cycle of 12/12 h, room temperature was kept at 24 ± 1 °C and 50 ± 5% relative humidity. Food (standard rat chow) and water were provided ad libitum throughout the experimental period. This study was conducted in strict accordance with the recommendations in the Guide for the Care and Use of Laboratory Animals of the National Institutes of Health [26]. All protocols were approved by the institutional review board for animal experiments of the Faculty of Medicine, Benha University, Egypt.

### 2.2. Experimental Design

After acclimatization for one week, study rats were randomly allocated into one of the following 4 groups: group 1 received a single intraperitoneal injection (i.p.) of 0.9% saline (control group, n = 10), group 2 received a single i.p. dose of cisplatin (5 mg/kg, Sigma–Aldrich, St. Louis, MO, USA) dissolved in 0.9% saline [27] (cisplatin group, n = 15), group 3 received i.p. cisplatin, and after 1 week, the rats received a single intravenous injection (i.v.) of 100 µL containing 1 × 10^6^ ADSCs (cisplatin + MSCs group, n = 15) or lastly, group 4 received cisplatin, and after 1 week, the rats received a single i.v. injection of 100 µL containing 1 × 10^6^ ADSCs preconditioned with 50 nM/L of rapamycin (cisplatin + MSCs + rapamycin group, n = 15). The i.v. injections were administered through the tail vein. Transplanted ADSCs were obtained from male rats such that the tracking of injected cells could be performed via detection of expression of male specific Sry gene in the liver of recipient females.

At the end of the experiment (30 days after cisplatin/saline injection), blood samples were collected from tail vein and left to clot and then centrifuged at 220× *g* for 10 min to obtain the serum, which was stored at −20 °C until measuring the liver biochemical parameters (AST, ALT and Albumin). Then, the rats were humanely euthanized via an overdose of inhalation general anesthesia, and the liver was immediately collected, and liver samples were obtained for further analyses.

### 2.3. Isolation and Culture of Adipose-Derived MSCs (ADSCs)

Adipose derived stem/stromal cells were isolated following previously published procedures [28,29]. Briefly, fat tissue samples obtained from male Sprague–Dawley rats were washed extensively with sterile Dulbecco’s phosphate buffered saline (D-PBS without calcium or magnesium) containing 3% Penicillin/streptomycin (Lonza, Switzerland) to remove contaminating blood cells. Then, fat tissue samples were minced using scissors and then subjected to digestion using 0.1% collagenase type I (Sigma, St. Louis, MO, USA) in a shaking water bath at 37 °C for 60 min (with gentle mixing by inverting every 15 min). Next, the digested samples were centrifuged at 1200× *g* for 5 min to obtain ADSCs containing cell pellet (stromal vascular fraction, SVF). The supernatant was discarded, and serum-free Dulbecco Modified Eagle Medium (DMEM) was added to the SVF, and the SVF cells were resuspended and centrifuged again; this washing step was repeated three times. Finally, the SVF was filtered through a sterile cell strainer (70 µm diameter, Greiner Bio-One GmbH, Frickenhausen, Germany), re-pelleted again by centrifugation, and finally resuspended in complete medium. The complete medium comprised Dulbecco’s Modified Eagle Medium (DMEM) with 4.5 g/L Glucose with L-Glutamine and sodium pyruvate (Lonza) containing 10% fetal bovine serum (FBS, Life Science Production, Barnet, UK), 1% Penicillin/streptomycin (Lonza). Cell number and viability were determined by trypan-blue exclusion. The isolated cells were plated at a density of 1 × 10^5^ cell/cm^2^ and cultured at 37 °C in a humidified atmosphere with 5% CO_*_. After 48–72 h, the complete medium was changed to remove non-adherent cells, and thereafter every three days. After cultured cells reached 80% confluency, the cells were detached with trypsin-EDTA (Lonza) and replated again. Third passage ADSCs were used in all the following experiments.

### 2.4. Characterization of Adipose MSCs by Flow Cytometry

Cultured ADSCs at passage three were characterized by flow cytometric evaluation of the expression of MSCs markers (CD29, CD44, CD105) with negative expression of hematopoietic and endothelial markers (CD45, CD34, CD31), this was performed following previously published protocols [24,30]. Briefly, ADSCs were detached, centrifuged to pellet the cells, washed twice, and fixed with 10% neutral buffered formalin for 30 min, washed again, and finally the cell pellet was resuspended in 100 µL FACS buffer containing primary antibody for 60 min at room temperature in the dark. The primary antibodies are FITC-labeled anti-CD29 (BioLegend, San Diego, CA, USA), anti-CD44 (BD Pharmingen, San Diego, CA, USA), anti-CD105 (abcam, San Diego, CA, USA), anti-CD45 (BioLegend, San Diego, CA, USA), anti-CD34 (BD Pharmingen, San Diego, CA, USA), and anti-CD31 (BD Pharmingen, San Diego, CA, USA). Then, propidium iodide was added to exclude dead cells, and the data was obtained on an Attune NxT Flow Cytometer (Applied Biosystems, Waltham, MA, USA). Twenty-thousand events were acquired, and the data was analyzed by Attune NxT Software v 3.1 (Applied Biosystems, Waltham, MA, USA). 

### 2.5. Rapamycin Preconditioning of ADSCs

Cultured ADSCs at passage three were either cultured with complete medium or complete medium containing 50 nM/L Rapamycin (LC Laboratories, Woburn, MA, Canada), and the cells were subsequently incubated for 2 h at 37 °C in a humidified atmosphere with 5% CO_*_ [25]. Then, the control medium or medium containing rapamycin was removed, ADSCs were washed with fresh medium (rapamycin free) twice and then the cells were trypsinized, washed twice and immediately used for further autophagy assessment, qPCR or transplantation experiments.

### 2.6. Assessment of Cell Viability by MTT Assay

ADSCs viability was evaluated using the MTT (3-[4,5-dimethylthiazol-2yl]-2,5-diphenyl-tetrazolium bromide) assay. Passage three ADSCs were cultured in a 96-well tissue culture plate and incubated for 24 h at 37 °C in a humidified atmosphere with 5% CO_*_. Then, the ADSCs were incubated with complete medium containing 0, 50, 75 or 100 nM/L rapamycin for two hours; three wells were selected for each concentration. Afterward, 10 μL MTT (5 mg/mL; Dojindo, Kumamoto, Japan) was added to each well, followed by additional incubation for four hours. Afterward, the medium was removed, and 100 μL DMSO was added to fully dissolve the generated formazan crystals. Finally, the absorbance was measured at 570 nm using FLUOstar^®^ Omega microplate reader (BMG LABTECH, Ortenberg, Germany).

### 2.7. Evaluation of Autophagic Activity 

#### 2.7.1. Transmission Electron Microscopy (TEM)

The formation of autophagosomes/autophagolysosomes within cells was directly observed in cultured ADSCs by using transmission electron microscopy as previously reported [25]; this step was performed in the Electron Microscopy Unit at Mansoura University. Briefly, ADSCs at passage three after preconditioning by rapamycin for two hours were detached, centrifuged to pellet cells (600× *g* for 10 min at room temperature), washed and centrifuged again. The cell pellet was then fixed using 2.5% glutaraldehyde for 24 h, postfixed with 1% osmium tetroxide for 2 h, and finally embedded in epoxy resin. Ultra-thin sections (60 nm) were obtained using ultramicrotome (RMC PT-XL PowerTome Ultramicrotome, Oberkochen, Germany) and the sections were mounted on copper grids and examined using a JEOL transmission electron microscope (JEOL JEM-2100, Tokyo, Japan).

#### 2.7.2. Immunostaining of Autophagosomes

In order to evaluate the formation of autophagosomes after rapamycin preconditioning, LC3 (microtubule-associated protein 1 light chain 3), a protein that is associated with autophagosomes membranes and considered as a marker for autophagosomes, was evaluated. Isolated ADSCs at passage three were incubated with 50 nM/L rapamycin for two hours in the incubator at 37 °C and 5% CO_*_. Afterward, the rapamycin-containing medium was aspirated and ADSCs were washed with PBS twice and then fixed with 10% neutral buffered formalin for 30 min, followed by permeabilization by Triton X-100 (0.5%) for 10 min. Then, the cells were washed twice and incubated with rabbit anti-LC3 antibody (1:200, ab48394, abcam, Waltham, MA, USA) overnight at 4 °C in a humidified chamber. Afterward, the primary antibody was aspirated, and the cells were washed twice, and then horseradish peroxidase (HRP)-labeled goat anti-rabbit secondary antibody was added (1:500, ab97051, abcam, Waltham, MA, USA), and the slides were incubated for 60 min at room temperature. The nuclei were counterstained with Mayer’s hematoxylin, and LC3-positive cells were examined under light microscopy.

#### 2.7.3. Gene Expression

To emphasize the induction of autophagy in ADSCs preconditioned by 50 nM/L rapamycin, RT-qPCR was performed to test the expression of autophagy markers LC3-II, p62 and mTOR genes.

### 2.8. Biochemical Analyses

Estimation of liver function was performed by quantifying the levels of aspartate transaminase (AST), alanine aminotransferase (ALT) and albumin in serum samples. These were tested in triplicate as we previously reported [31] using commercially available kits and following manufacturer’s protocol.

### 2.9. Evaluation of Liver Oxidative Stress and Antioxidant Parameters

Immediately post-sacrifice samples from the liver tissues were collected, washed twice in PBS + 0.16 mg/mL heparin, homogenized and centrifuged, as we previously reported [31,32]. The level of liver lipid peroxidation was assessed by measuring the levels of malonyl dialdehyde (MDA). For evaluating the levels of antioxidant enzymes in the liver, superoxide dismutase (SOD) and glutathione peroxidase (GPX) were measured. The assessment of MDA, SOD and GPX levels was performed using commercial kits (Bio-diagnostics Co., Cairo, Egypt and BioVision, Inc., Milpitas, CA, USA), after the manufacturer recommendations, and in triplicates.

### 2.10. Histopathological Studies 

The preparation of liver tissue samples and histopathological examinations were performed as we previously reported [31]. In brief, obtained liver samples were immediately washed in warm saline and fixed using 10% neutral buffered formalin solution. After fixation, the samples were dehydrated by serial ascending concentration of alcohol (BDH, UK), and xylene (BDH, UK), and then were embedded in paraffin wax (Sheruood, Sherwood, OR, USA). Paraffin-embedded liver tissue samples were cut into 5 µm sections by microtome (Mainz, West Germany). The sections were mounted on glass slide and stained with standard hematoxylin and eosin stain (or immunostaining as in the next step). The extent of liver damage and any lesions were blindly assessed by an experienced pathologist. The slides were examined under light microscopy (Zeiss, Jena, Germany). 

### 2.11. Immunostaining

Liver tissue sections (5 µm) were deparaffinized, rehydrated and underwent heat epitope antigen retrieval by boiling at approx. 100 °C for 10 min in preheated 0.01 M citrate buffer (pH 6) using a microwave. Blocking of endogenous peroxidases was performed by incubation with 3% H_*_ O_*_ for 10 min and nonspecific binding was blocked by incubation with 5% goat serum for 20 min at room temperature. The slides were incubated with primary antibodies, rabbit anti-TGF beta 1 antibody (1:250, ab215715, abcam, Waltham, MA, USA) and rabbit Anti-SDF-1 (1:150, Catalog# A00053, Boster Bio, Pleasanton, CA, USA) in a humidified immunostaining chamber overnight at 4 °C. Then, the excess primary antibody was removed, slides were washed several times, the HRP-labeled goat anti-rabbit secondary antibody was added (1:500, ab97051, abcm, Waltham, MA, USA), and the slides were incubated for 1 h at room temperature. Then, visualization of bound antibodies was performed using DAB (3,3′-Diaminobenzidine) substrate kit, and Mayer’s hematoxylin was used as a counterstain to visualize the nuclei. Five sections were randomly selected from each group and evaluation of the intensity of staining was performed with ImageJ software (v 1.53, National Institutes of Health, Bethesda, MD, USA).

### 2.12. Gene Expression Analysis

Total RNA from liver tissue samples (100 mg) was extracted using TRIzol™ Reagent (Invitrogen, CA, USA) following manufacturer instructions. The rt-qPCR was performed following standard procedures as we previously reported [33]. The total RNA obtained was checked for concentration and purity at 260 and 280 nm wavelengths using nanodrop (Quawell Q5000, Quawell Technology, Inc., San Jose, CA, USA). Then, reverse transcription of the extracted mRNA into cDNA was performed using RevertAid First Strand cDNA Synthesis Kit (Thermo Scientific, Cat. No. 1622).

The expression levels of mRNA were quantified using StepOnePlus™ Real-Time PCR system (Applied Biosystems, Waltham, MA, USA) by using QuantiTect SYBR^®^ Green PCR Kit (Qiagen, Cat. No. 204141). Quantitative real-time PCR (qRT-PCR) for the genes of interest was conducted by using the primers listed in Table 1 and were normalized against GABDH (as a housekeeping gene); the calculations followed the 2^−ΔΔCt^ method as previously described.

### 2.13. Statistical Analysis

The obtained data from this study was analyzed by one-way ANOVA, followed by least significant difference (LSD) analysis to compare the difference between study groups. The statistical analyses were performed using PASW statistical package (SPSS v18, SPSS Inc., Chicago, IL, USA). Results are expressed as mean ± standard deviation. Statistical significance was considered when *p* values were ≤ 0.05.

## 3. Results

### 3.1. Characterization of Isolated ADSCs

**Figure 1:**
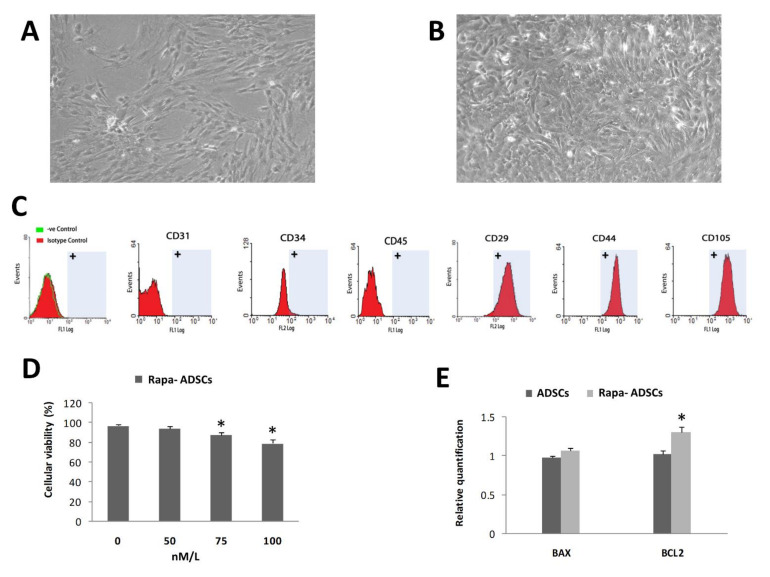
Effect of rapamycin preconditioning for 2 h on ADSCs. (**A**) Typical fibroblast-like morphology of cultured ADSCs. (**B**) Cultured ADSCs with 50 nM/L rapamycin. (**C**) Surface characterization of different stem cells markers on isolated ADSCs by flow cytometry. (**D**) Effects of different concentrations of rapamycin, 0, 50, 75 and 100 nM/L, on ADSCs viability. (**E**) Effect of 50 nM/L rapamycin on expression of BAX and BCL2 in non-preconditioned and preconditioned ADSCs. Statistical significance, * = *p* < 0.05.

Isolated ADSCs showed typical culture morphology of attached stem/stromal cells (Figure 1A); i.e., typical spindle-shaped plastic adherent cells. These cells were characterized at passage three using flow cytometric analysis (Figure 1C); there was positive expression of specific mesenchymal stem cell markers (CD29, CD44, CD105) and negative expression of hematopoietic and endothelial markers (CD45, CD34, CD31). 

### 3.2. Effect of Rapamycin Preconditioning on Viability of Cultured ADSCs

Rapamycin preconditioning of ADSCs for two hours did not affect the cell morphology (Figure 1B) or flow cytometry characteristics of cultured ADSCs. By measuring cellular viability using MTT assay (Figure 1D), the results showed that increasing the concentration of rapamycin to 75 and 100 nM/L significantly (*p* < 0.05) reduced the viability of ADSCs to 86% and 79%, respectively, while the 50 nM/L concentration did not significantly affect ADSCs viability, and therefore, this concentration (50 nM/L) was used in all experiments hereafter. The gene expression of BAX gene was insignificantly increased, while BCL2 was significantly increased (*p* < 0.01) in rapamycin-preconditioned ADSCs compared to normal ADSCs (Figure 1E).

### 3.3. Effect of Rapamycin on Autophagy of ADSCs

**Figure 2:**
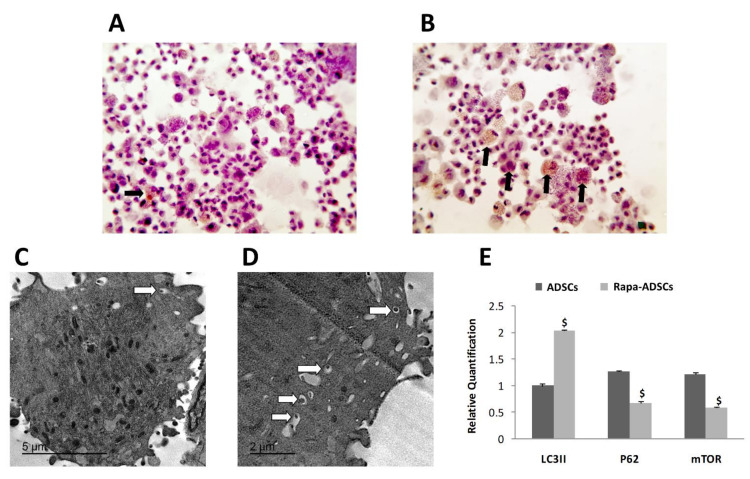
The effect of rapamycin preconditioning on autophagosomes formation in ADSCs. (**A**,**B**) Autophagosomes are immunohistochemically labeled with LC3II antibodies; magnification 400×. Black arrows indicate LC3II-positive autophagosomes inside ADSCs. (**A**) Non-preconditioned ADSCs, (**B**) Rapamycin (50 nM/L) preconditioned ADSCs. (**C**,**D**) The effect of rapamycin preconditioning on autophagic ultrastructures; white arrows indicate autophagosomes. (**C**) Non-preconditioned ADSCs, (**D**) Rapamycin (50 nM/L)-preconditioned ADSCs. (**E**) Effects of 50 nM/L rapamycin on expression of LC3II, p62 and mTOR genes in non-preconditioned and preconditioned ADSCs. Statistical significance, $ = *p* < 0.01.

Enhanced autophagy activation in ADSCs was visualized by immunohistochemical staining of LC3-II-positive autophagosomes, where more autophagosomes were seen in ADSCs preconditioned with rapamycin compared to control non-preconditioned ADSCs (Figure 2B compared to Figure 2A). Similarly, TEM examination of the ultrastructure of ADSCs confirmed the presence of more autophagosomes with larger size inside the preconditioned ADSCs compared to normal ADSCs (Figure 2D compared to Figure 2C).

Furthermore, in ADSCs preconditioned with 50 nM/L rapamycin, the gene expression of autophagosome specific gene LC3II was significantly higher, while the expression of ubiquitin-binding protein p62, associated with protein degradation and of mTOR genes was significantly lower compared to ADSCs that were not preconditioned (*p* < 0.01, Figure 2E).

### 3.4. Detection of Administered ADSCs in the Liver of Recipient Animals

The expression of similar levels of *Sry* gene was detected in the liver of recipient females in both groups 3 and 4 (receiving naïve ADSCs or rapamycin-preconditioned ADSCs), confirming the translocation of male-derived ADSCs into the injured livers of recipient females.

### 3.5. Effects of Rapamycin-Preconditioned ADSCs on Liver Function 

**Figure 3:**
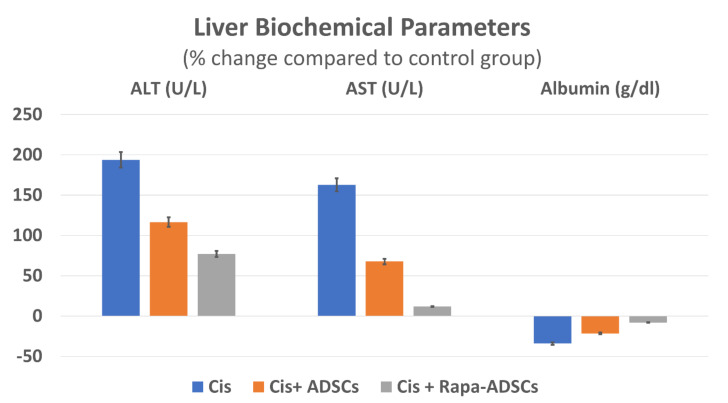
The effect of administering rapamycin-preconditioned ADSCs on liver biochemical parameters, AST, ALT and albumin in cisplatin (Cis)-induced liver damage. Cis, cisplatin (i.p. injection of 5 mg/kg in 0.9% saline) group; Cis + ADSCs, cisplatin group receiving ADSCs (one million cells, intravenous in tail vein); Cis + Rapa-ADSCs, cisplatin group receiving same number of ADSCs preconditioned with rapamycin (50 nM/L, 2 h prior to administration).

Levels of AST, ALT and albumin in control group fell within normal range reported for lab animals [34,35]. Cisplatin administration led to significant (*p* < 0.01) increase of serum levels of ALT and AST, while albumin levels decreased compared to the control group. Following ADSCs injection, serum levels of AST and ALT were significantly (*p* < 0.01) reduced, by 25 and 36%, respectively, while albumin levels were increased significantly (*p* < 0.01) by 19% compared to cisplatin group. Importantly, group 4, receiving rapamycin preconditioned ADSCs showed the best results, wherein the least differences in AST, ALT and albumin levels from the control group were noticed in this group. This is compared to other cisplatin groups (groups 2 and 3 receiving cisplatin alone or cisplatin + ADSCs). Serum levels of different liver biochemical parameters in response to cisplatin, ADSCs or rapamycin-ADSCs are shown in Table 2 and Figure 3.

### 3.6. Effects of Rapamycin-Preconditioned ADSCs on Liver Antioxidant Profile

**Figure 4:**
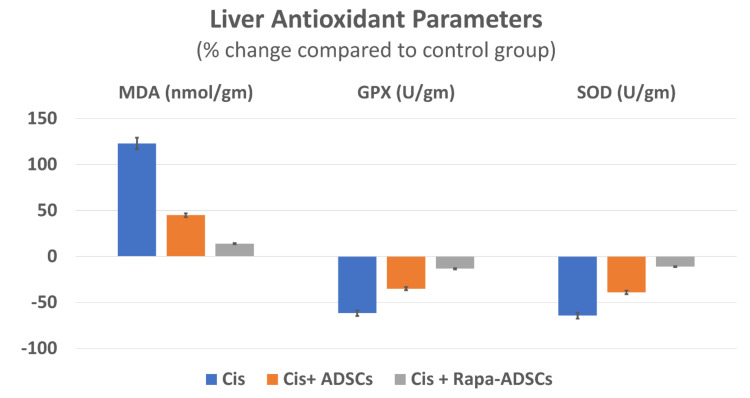
The effect of administering rapamycin-preconditioned ADSCs on liver antioxidant parameters, MDA, GPX and SOD, in cisplatin (Cis)-induced liver damage. Cis, cisplatin (i.p. injection of 5 mg/kg in 0.9% saline) group, Cis + ADSCs, cisplatin group receiving ADSCs (one million cells, intravenous in tail vein) and Cis + Rapa-ADSCs, cisplatin group receiving same number of ADSCs preconditioned with rapamycin (50 nM/L, 2 h prior to administration).

Following cisplatin injection, higher levels of lipid peroxidation marker (MDA) in liver tissue were obtained with lowest levels of antioxidant enzymes (GPX and SOD) (*p* < 0.01). Administration of ADSCs resulted in improvement in liver antioxidant profile, where significantly lower MDA levels (by 35%) and significantly higher GPX and SOD levels (by 70 and 71%, respectively) were obtained. These effects were further improved following administration of rapamycin preconditioned ADSCs (MDA was reduced by 48% and GPX and SOD increased by 126 and 150%, compared to cisplatin group, *p* < 0.05). Different liver antioxidant parameters in response to cisplatin, ADSCs or rapamycin-ADSCs are shown in Table 3 and Figure 4.

### 3.7. Effects of Rapamycin-Preconditioned ADSCs on Liver Histopathological Picture

**Figure 5:**
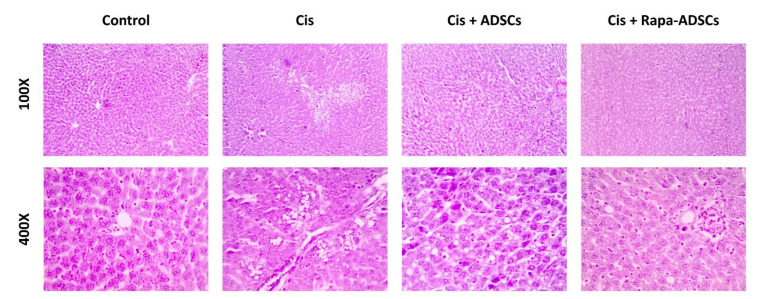
Photomicrographs showing the effect of administering rapamycin-preconditioned ADSCs on liver histopathological picture in cisplatin (Cis)-induced liver damage. Cis, cisplatin (i.p. injection of 5 mg/kg in 0.9% saline) group, Cis + ADSCs, cisplatin group receiving ADSCs (one million cells, intravenous in tail vein) and Cis + Rapa-ADSCs, cisplatin group receiving same number of ADSCs preconditioned with rapamycin (50 nM/L, 2 h prior to administration). In the control group, normal liver histological picture was evident. In the cisplatin group, the liver showed marked degenerative and inflammatory changes, including distorted shape of hepatic lobules, inflammatory cells infiltration, necrotic hepatocytes and extensive vacuolations. In the cisplatin group that received ADSCs, the histopathological picture was improved with partially restored hepatic architecture and few degenerative lesions and vacuolations. Finally, in the cisplatin group receiving rapamycin preconditioned ADSCs, the best histological improvements were achieved in the liver compared to other groups, with near normal hepatic microscopic structure.

Normal liver histological picture was seen in control group, hexagonal lobules with central vein and the normal hepatocytes, which appeared polygonal in shape, and sinusoids containing Kupffer cells. In cisplatin group, degenerative changes were seen in the liver with several hepatocytes showing necrotic changes as pyknotic nuclei and strongly acidophilic cytoplasm. Dilated sinusoids and infiltrating inflammatory blood cells in addition to vacuolations in hepatocytes were also seen. Treatment with ADSCs resulted in an improved histopathological picture with less degenerative changes in hepatocytes, less prominent vacuolations, and reduced leukocytic infiltration compared to the cisplatin group, whereas the best improvement in liver histopathological picture was seen in group 4 receiving rapamycin-preconditioned ADSCs, where remarkable reduction in cisplatin-induced pathological lesions was noticed. Different liver histopathological pictures in response to cisplatin, ADSCs or rapamycin-ADSCs are shown in Figure 5.

### 3.8. Effects of Rapamycin-Preconditioned ADSCs on Liver TGF-β1/Smad Signaling Pathway

**Figure 6:**
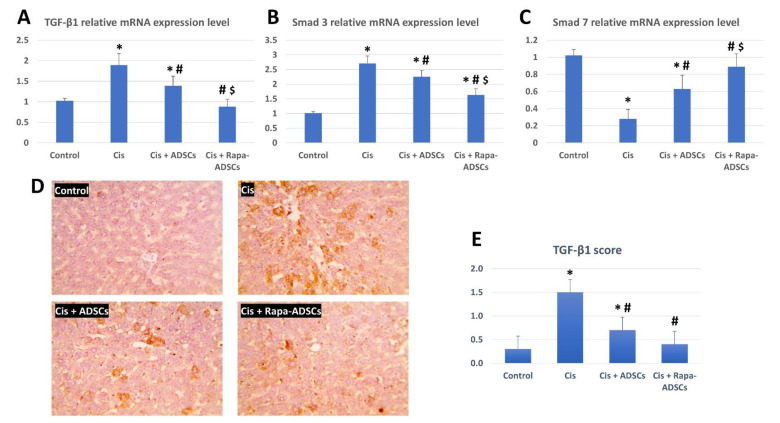
The effects of administering rapamycin-preconditioned ADSCs on liver TGF-β1/Smad signaling pathway in cisplatin (Cis)-induced liver damage. (**A**) TGF-β1 relative mRNA expression in the study groups. (**B**) Smad3 relative mRNA expression in the study groups. (**C**) Smad7 relative mRNA expression in the study groups. (**D**) Immunohistochemical staining of TGF-β1 in the liver tissue in the different groups, magnification 400×. (**E**) TGF-β1 score from the images in (**D**) from the different studied groups. Cis, cisplatin (i.p. injection of 5 mg/kg in 0.9% saline) group; Cis + ADSCs, cisplatin group receiving ADSCs (one million cells, intravenous in tail vein); Cis + Rapa-ADSCs, cisplatin group receiving same number of ADSCs preconditioned with rapamycin (50 nM/L, 2 h prior to administration). Statistical significance, * vs. control group (*p* < 0.01), # vs. Cis group (*p* < 0.01) and $ vs. Cis+ADSCs group (*p* < 0.05).

The expression of TGF-β1, Smad3 and Smad7 mRNA was evaluated in the study groups, and results are shown in Figure 6. Cisplatin administration led to significant upregulation of expression levels of TGF-β1 and Smad3 genes and significantly downregulated the expression of Smad7 gene (*p* < 0.01). Following ADSCs treatment, the effects of cisplatin were partially alleviated with reduced expression of TGF-β1 and Smad3 genes and partially restored expression of Smad7, while after administration of rapamycin-preconditioned ADSCs, the expression level of TGF-β1, Smad3 and Smad7 was considered improved compared to other cisplatin groups and approached the control group levels.

### 3.9. Effects of Rapamycin-Preconditioned ADSCs on Liver PI3K-AKT Signaling Pathway and NF-κB Gene Expression

**Figure 7:**
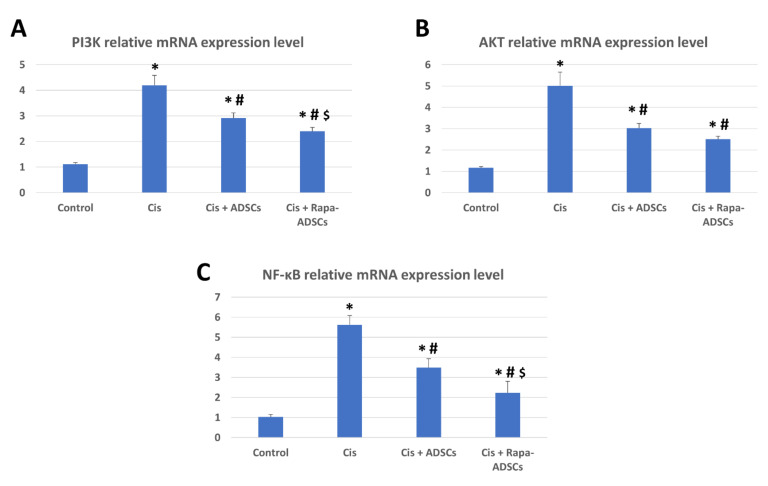
The effects of administering rapamycin-preconditioned ADSCs on liver PI3K-AKT signaling pathway and NF-κB gene expression in cisplatin (Cis)-induced liver damage. (**A**) PI3K relative mRNA expression in the study groups. (**B**) AKT relative mRNA expression in the study groups. (**C**) NF-κB relative mRNA expression in the study groups. Cis, cisplatin (i.p. injection of 5 mg/kg in 0.9% saline) group; Cis + ADSCs, cisplatin group receiving ADSCs (one million cells, intravenous in tail vein); Cis + Rapa-ADSCs, cisplatin group receiving same number of ADSCs preconditioned with rapamycin (50 nM/L, 2 h prior to administration). Statistical significance, * vs. control group (*p* < 0.01), # vs. Cis group (*p* < 0.01) and $ vs. Cis+ADSCs group (*p* < 0.05).

The relative expression of PI3K, AKT and NF-κB mRNA was significantly increased (4, 5 and 5.5 times, respectively, *p* < 0.01, compared to their control levels) following cisplatin administration (Figure 7). Following ADSCs treatment, the expression levels of PI3K and AKT were significantly reduced (*p* < 0.05). However, this reduction was best achieved in the rat group receiving rapamycin preconditioned ADSCs.

### 3.10. Effects of Rapamycin-Preconditioned ADSCs on Liver SDF-1α Gene Expression and Levels of SDF-1α Protein in the Liver

**Figure 8:**
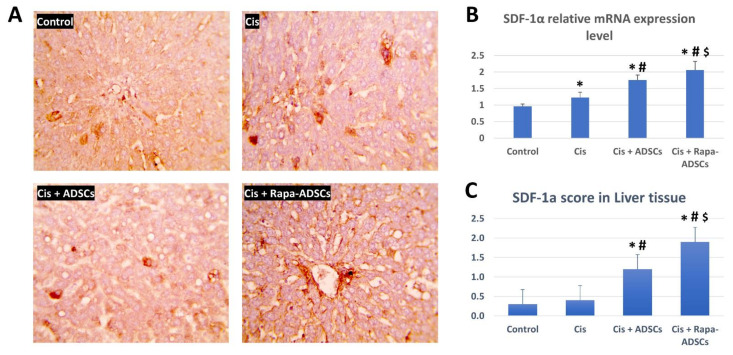
The effects of administering rapamycin-preconditioned ADSCs on liver SDF-1α gene expression and liver tissue levels in cisplatin (Cis)-induced liver damage. (**A**) Immunohistochemical staining of SDF-1α in the liver tissue in the different groups, magnification 400×. (**B**) SDF-1α relative mRNA expression in the study groups. (**C**) SDF-1α score from the images in (**A**) from the different studied groups. Cis, cisplatin (i.p. injection of 5 mg/kg in 0.9% saline) group; Cis + ADSCs, cisplatin group receiving ADSCs (one million cells, intravenous in tail vein); Cis + Rapa-ADSCs, cisplatin group receiving same number of ADSCs preconditioned with rapamycin (50 nM/L, 2 h prior to administration). Statistical significance, * vs. control group (*p* < 0.01), # vs. Cis group (*p* < 0.01) and $ vs. Cis+ADSCs group (*p* < 0.05).

The levels of SDF-1α mRNA and protein expression in the liver were evaluated in the different study groups, and the results are shown in Figure 8. The mRNA expression of SDF-1α gene was not significantly increased in the cisplatin group, but it was significantly increased in the group receiving ADSCs, and the highest levels were obtained in the group receiving rapamycin-preconditioned ADSCs (*p* < 0.01. Figure 8B). Additionally, the tissue levels of SDF-1α were immunohistochemically evaluated in liver sections of the different study groups (Figure 8A,C). The mean number and overall area of the SDF-1α positive cells in the liver were also significantly higher in the group receiving ADSCs, while the highest SDF-1α levels were in the group receiving rapamycin-preconditioned ADSCs.

## 4. Discussion

Here, we show that rapamycin-preconditioning of ADSCs increased their autophagic activity, and subsequently, enhanced their therapeutic potential following injection in a rat model of liver injury caused by cisplatin. First, rapamycin activation of autophagy in ADSCs was assessed through the evaluation of various markers. The most prominent was the increase in LC3-II positive autophagosomes inside ADSCs, increase in the number of autophagosomes seen via TEM, upregulation of LC3-II gene and downregulation of p62 and mTOR genes, and consequently, enhanced survival of rapamycin-preconditioned ADSCs and reduced apoptosis via upregulation of the antiapoptotic BCL2 gene. 

With respect to biogenesis of autophagosomes in ADSCs, one of the important microtubule-associated protein 1 light chain 3 (LC3) types that is associated mainly with autophagosome formation is LC3-II, which is membrane bound and specifically labels autophagosome membranes, and its quantity is specifically positively correlated with the amount of autophagosomes [36]. In addition, p62 (also known as Sequestosome-1 or ubiquitin-binding protein p62), is a protein that is responsible for the ubiquitination of important cellular proteins and is associated with various cellular processes including NF-κB signaling, apoptosis and ubiquitin-mediated autophagy [37,38]. It was found essential for the autophagic degradation of p62-associated ubiquitinated proteins [39]. In this study, rapamycin-preconditioned ADSCs had reduced mTOR expression, reduced p62 expression, higher expression of LC3-II gene and higher number of LC3-II positive autophagosomes, all indicating the activation of autophagy in ADSCs with reduction of both p62-mediated protein degradation and apoptosis rate.

It is well established that mTOR is in the center of multiple signaling pathways where various extracellular and intracellular signals meet and become integrated for the organization of cellular metabolism and growth, and it is frequently dysregulated in metabolic disorders and cancer [40]. One of the known TOR complexes is TOR complex 1 (TORC1), which is sensitive to rapamycin in nanomolar levels; once rapamycin binds to the FKBP–rapamycin binding domain of TOR it leads to the inhibition of TORC1, and thus, leads to controlled cellular growth and promoted autophagy [40]. In addition, the inhibition of TORC1 can activate TORC2, another mTOR complex, but is insensitive to rapamycin, which in turn can activate AKT and promote anti-apoptotic effects [41]. Previous reports also highlighted that rapamycin activates autophagy, reduces apoptosis and enhances the survival and differentiation of bone marrow MSCs [25,42,43]. Thus, rapamycin both enhances autophagy and reduces apoptosis, which was observed in the present study where rapamycin preconditioned ADSCs had a lower mTOR expression and higher autophagic activity than non-treated ADSCs. The broadly used dose of rapamycin for activating autophagy in cells (i.e., 50 nM/L) did not lead to adverse effects on ADSCs in this study. However, increasing rapamycin concentration more than 75 nM/L was detrimental to ADSCs survival, indicating that higher doses of rapamycin do not necessarily indicate beneficial effects and better survival. A possible explanation can be derived from previous observations in MSCs (and most probably in other cells as well), where excessive autophagy activation can lead to cellular death [44].

Following evaluation of autophagy activation, ADSCs with enhanced autophagy (via rapamycin-preconditioning) in this study were then tested for their therapeutic potential in a rat model of liver injury induced by cisplatin, and this was compared to non-preconditioned ADSCs. The liver histopathological picture and biochemical and antioxidant profiles were improved following administration of rapamycin preconditioned ADSCs. In addition, in the liver, the gene expression of NF-κB and SDF-1α genes and components of the TGF-β1/Smad and PI3K-AKT-mTOR signaling pathways all approached near-normal levels with only slight differences compared to the normal control group. Finally, immunohistochemically, the liver levels of TGF-β were reduced to comparable levels to the control group while SDF-1α expression reached maximum levels in the liver of the cisplatin group receiving rapamycin-preconditioned ADSCs.

TGF-β1 is one member of the TGF-β superfamily that is concerned with various functions during normal development, including cell proliferation, differentiation, and migration, and plays various roles in pathologic development such as cancer [45]. TGF-β members also play important roles in tissue stem cells maintenance and differentiation [46], and loss of tumor suppressor effects or stimulation of tumor promotor effects of TGF-β signaling induce cancer stem cells formation and progression of hepatocellular carcinoma [47]. Smad 3 is known to be an important signaling molecule for TGF-β signaling pathway, while Smad7 inhibits TGF-β/Smad signaling [45]. More specifically, in the liver, an increase in expression of TGF-β1, and members of TGF-β family in general, was related to the pathogenesis and progression of all stages of liver diseases [48,49]. It was noted that TGF-β1 is usually associated with the development of fibrosis not only in the liver but also in other organs such as the lungs, heart and kidneys [50,51,52]. The blockage of TGF-β pathway is considered as an effective strategy for treating liver diseases [53,54,55]. In another model of liver injury induced by carbon tetrachloride, higher levels of TGF-β1 in liver tissue and activated TGF-β/Smad pathway were indicators for severe liver injury [51]. In addition, the latter study demonstrated that the overexpression of Smad3 in injured livers further aggravated the condition and induced more liver damage, more apoptosis, more inflammation and more serum concentrations of AST, ALT and TGF-β itself. The current results showed that, following cisplatin administration, the expression levels of TGF-β1 and Smad3 were increased and Smad7 expression was decreased, indicating the occurrence of pathologic liver damage. This was further confirmed by the prominent damaged histopathologic picture and increased TGF-β1 levels in liver tissue. The administration of ADSCs reduced liver damage via attenuating TGF-β1 and Smad3 and increasing Smad7 gene expression, which is a suppressor to TGF-β, together with reducing the tissue levels of TGF-β1 and increasing SDF-1α levels in the liver. This led to reduced liver tissue damage seen by histopathologic examination and associated serum biochemical (AST, ALT and albumin) and antioxidant (MDA, GPX and SOD) parameters. However, the administration of ADSCs with activated autophagy via rapamycin-preconditioning resulted in better improvements in serum biochemical, liver histopathological, liver antioxidant and liver TGF-β/Smad genes expression profiles, all reaching to near normal levels. In addition, the administration of ADSCs with activated autophagy resulted in lower tissue levels of TGF-β1 and the highest levels of SDF-1α in liver tissue.

As highlighted above, mTOR is a key signaling molecule that is responsible for maintaining cell growth, apoptosis and autophagy in a wide range of body cells, and its upstream activators are PI3K and AKT (details about PI3K-AKT-mTOR signaling pathway are reviewed elsewhere [56]). In addition, the upregulation of PI3K-AKT-mTOR signaling pathway in necrotic cell lines suppresses autophagy and promotes cell death [57], while the reduction of PI3K and AKT gene expression in the liver following administration of ADSCs is a good indicator for their beneficial therapeutic effects. This is based on the previous observations that downregulation of elevated PI3K-AKT-mTOR signaling, via miR-101 [58], lncRNA GAS5 and miR-23a [59] or miR-29a [60], resulted in reducing the extent of chronic liver damage by decreasing hepatic stellate cells activation and liver fibrosis. Similarly, in the kidney, a recent report showed that cisplatin-induced kidney damage and associated inflammatory responses were reduced following maltol pretreatment which reduced the activated PI3K-AKT signaling pathway [61]. Apparently, the down regulation of PI3K-AKT-mTOR signaling is also crucial for the recovery following cisplatin administration and for improving cisplatin sensitivity in hepatocellular carcinoma [62], lung cancer [63,64], gastric cancer [65,66], and cervical cancer [67]. In contrast, autophagy inhibition in hepatocellular carcinoma cells led to the upregulation of PI3K-AKT-mTOR signaling pathway and promoted the proliferation, migration and metastasis of hepatocellular carcinoma cells [68].

Following cisplatin administration in the current study, gene expression levels of PI3K and AKT were elevated indicating activated PI3K-AKT-mTOR signaling. However, their levels fell after the administration of ADSCs, but were still significantly higher than the control group. These elevated levels of PI3K and AKT are not ideal for liver regeneration as discussed above, whereas the lowest expression levels of PI3K and AKT were detected in the cisplatin group receiving autophagy stimulated ADSCs, still again, their levels were somewhat higher than the control group. It is not clear why there were slightly higher than basal levels of the gene expression of PI3K and AKT in the cisplatin group receiving rapamycin preconditioned ADSCs. A possible explanation can be derived from previous studies, which showed that the direct inhibition of PI3K-AKT-mTOR signaling via administration of rapamycin alone resulted to be not beneficial in clinical trials since several side effects were recorded, including hepatotoxicity and liver damage [69], probably due to blocked mTOR and its linked cell growth and metabolic functions. This can justify our observation that modestly higher levels of PI3K-AKT-mTOR signaling in rapamycin-preconditioned ADSCs group (but not excessively higher as in cisplatin alone group) is necessary to promote liver healing and hepatocytes’ growth to reduce the extent of liver damage, while, probably, basal levels of PI3K-AKT-mTOR signaling (as in the control group) will not promote speedy recovery and regeneration of the damaged liver. Therefore, rapamycin-preconditioning of stem cells combines the benefits of using both rapamycin and stem cells, while providing a safer alternative than using rapamycin alone for various therapeutic applications. This was seen in the present study; however, more research is required to confirm this combined beneficial effect.

The transcription factor NF-κB has complex functions and was found to be essential for inflammatory responses affecting cell proliferation, migration, and apoptosis, and in general, is a key linker between inflammation and cancer (reviewed in, [70,71]). The expression of NF-κB in the liver is an indication to the extent of inflammation and initiation of fibrotic reactions and higher levels indicate poor prognosis in liver pathologies and predispose to cancer development [72]. Upon activation of NF-κB in the liver, hepatic stellate cells attract more Kupffer cells into the injured areas, Kupffer cells in turn produce TGF-β, which via TGF-β/Smad signaling activate more hepatic stellate cells, and the end result is highly activated hepatic stellate cells and more extracellular matrix production, which further promotes fibrosis and liver injury [72]. The selective inhibition of NF-κB in the liver, using synthetic “decoy” NF-κB, was beneficial to reduce the extent of liver damage induced by carbon tetrachloride; in addition, this reduced the development of fibrosis and allowed the liver to regenerate [73]. In the present study, NF-κB expression in the liver was increased following cisplatin administration, indicating an activated immune system seen by more leukocytic infiltration in the liver tissue, and liver damage was evident. Following administration of ADSCs, the levels of NF-κB were reduced but still were higher than the control group. However, in the rapamycin-preconditioned ADSCs group, the best reduction in the levels of NF-κB were detected, indicating reduced inflammatory condition, least leukocytic infiltration, and better liver healing and regeneration seen by histology. In addition, reduced NF-κB in the rapamycin-preconditioned ADSCs group also means less activated hepatic stellate and Kupffer cells and reduction in their activation by TGF-β/Smad signaling, where the lowest levels of TGF-β and lower TGF-β/Smad signaling in liver tissue was seen in this group. Our results showed that, without the need to introduce synthetic factors, the expression of NF-κB can be reduced by administering rapamycin-preconditioned ADSCs.

Liver sinusoidal epithelial cells were recently shown to be key players in liver injury and that they are associated with the development of fibrosis [74]. Following liver injury, the levels of SDF-1α increase significantly inside the injured regions; this is important to attract stem cells [75] and macrophages [76] to initiate tissue repair processes in the injured liver. Inhibiting SDF-1α receptor (also called C-X-C chemokine receptor-4; CXCR4) using AMD3100 (a CXCR4 antagonist) led to delayed regeneration of liver damage and an increase in proinflammatory reactions, which further contributed to impairing liver repair, while the administration of SDF-1 led to enhanced liver regeneration [76]. In addition, hepatic oval cells were found to migrate to injured liver regions along an SDF-1α gradient, suggesting that SDF-1α/CXCR4 interaction is essential for liver repair following injury [77]. The mechanism of SDF-1α upregulation as a response to facilitate and guide tissue healing is not exclusive to the liver since it was also found in various organs as skin [78], kidney [79], and in the migration and homing of endogenous or transplanted stem cells to damaged tissues, as ischemic brain lesions [80,81]. Therefore, increased SDF-1α was described to play a vital role in stem/progenitor cells migration toward injured tissues to initiate various repair mechanisms [82]. In the present study, the levels of SDF-1α reached its highest levels in the rat group receiving rapamycin-preconditioned ADSCs and SDF-1α expression was seen localized in sinusoidal endothelial cells and around the central vein in the cisplatin-injured liver. Conversely, non-preconditioned ADSCs fell below in terms of SDF-1α expression in the liver. This highlights that a higher liver repair capacity was achieved by administration of rapamycin-preconditioned ADSCs following cisplatin-induced liver damage. 

Another important point is that autophagy, in general, plays an important role in liver homeostasis and tissue repair. In support, it was shown that impaired autophagy in liver cells, for example in sinusoidal endothelial cells, increases oxidative stress and liver fibrosis, and thus autophagy was confirmed as an important process that maintains liver endothelial cells homeostasis [83]. Stimulation of autophagy in ADSCs via rapamycin preconditioning can also stimulate autophagy in neighboring liver cells at the injury site via the various paracrine mechanisms of stem cells. In a recent study with bone marrow MSCs, autophagy activation via rapamycin was shown to stimulate the release of pro-survival factors (such as HGF, IGF-1, SCF, SDF-1, VEGF, HIF-1α and IL-10), which, via paracrine effects, contributed to myocardial repair and regeneration following administration of these rapamycin-treated MSCs in infarcted myocardium [25]. However, the transfer of autophagy to neighboring liver cells was not investigated in the present study.

## 5. Conclusions

In conclusion, the stimulation of autophagy in ADSCs not only increased their survival and reduced their apoptosis, but also increased their therapeutic potential in case of liver injury induced by cisplatin. The effects were via the modulation of components of the TGF-β1/Smad and PI3K-AKT-mTOR signaling pathways and reducing NF-κB gene expression, in addition to reducing TGF-β and increasing SDF-1 levels in liver tissue and enhancing the histopathological and serum biochemical parameters associated with liver damage. Furthermore, the results of the present study demonstrated that the administration of ADSCs with activated autophagy via rapamycin preconditioning showed superior results compared to normal non-preconditioned ADSCs, highlighting the importance of autophagy in potentiating the favorable effects of stem cell-based therapy. Future studies are needed to detect whether this favorable therapeutic effect is caused by autophagy-stimulated ADSCs alone, or in addition, pro-survival autophagic effect and factors were transferred from autophagy-stimulated ADSCs to damaged liver cells via the known stem cells’ paracrine effects stimulating their survival and reducing liver damage.

## Figures and Tables

**Table 1:** Primers used for quantitative RT-PCR.

Gene Name	Sequence (5′-3′)	Accession No.
LC3	F- CCAGGAGGAAGAAGGCTTGG R- GAGTGGAAGATGTCCGGCTC	NM_022867.2
P62	F- TGCTCCATCAGAGGATCCCA R- TTTCTGCAGAGGTGGGTGTC	NM_175843.4
mTOR	F- TTGTGTCCTGCTGGCTG AAC R- GCTCTTTGTAGTGTAGTGCTTTGG	NM_019906.2
BAX	F: GGCGATGAACTGGACAACAA R: CAAAGTAGAAAAGGGCAACC	NM_017059.2
BCL2	F: GGTGAACTGGGGGAGGATTG R: GCATGCTGGGGCCATATAGT	NM_016993.1
NF-κB	F- GGACAGCACCACCTACGATG R- CTGGATCACTTCAATGGCCTC	NM_001276711.1
TGFβ1	F- CACTCCCGTGGCTTCTAGTG R- GGACTGGCGAGCCTTAGTTT	NM_021578.2
SDF1α	F- GAGCCATGTCGCCAGAGCCAAC R- CACACCTCTCACATCTTGAGCCTCT	NM_001033882.1
PI3K	F- TCTCCGTAGCGGGGCACCAG R- AACCAGCCAATATCTTCTGG	XM_017590649.2
AKT	F- GAGGAGGAGACGATGGACTTC R- GGCATAGTAGCGACCTGTGG	NM_033230.3
Smad3	F- AGGGCTTTGAGGCTGTCTACC R- ACCCGATCCCTTTACTCCCA	NM_013095.3
Smad7	F- GGGGGAACGAATTATCTGGC R- CGCCATCCACTTCCCTTGT	NM_030858.2
Sry	F- TGGGACTGGTGACAATTGTC R- GAGTACAGGTGTGCAGCTCT	NM_012772
GAPDH	F- AGACAGCCGCATCTTCTTGT R- TTCCCATTCTCAGCCTTGAC	NM_017008.4

**Table 2:** The impact of rapamycin-preconditioned ADSCs on liver biochemical parameters; ALT, AST and albumin, after cisplatin (Cis)-induced liver damage.

Group	ALT (U/L)	AST (U/L)	Albumin (g/dL)
Control	48.16 ± 3.06	102 ± 14.71	3.35 ± 0.46
Cis	141.17 ± 8.56 *	268.67 ± 54.7 *	2.21 ± 0.61 *
Cis + ADSCs	104.33 ± 10.17 *^,#^	171 ± 71.35 *^,#^	2.63 ± 0.61 *^,#^
Cis + Rapa-ADSCs	85.83 ± 10.57 *^,#,$^	114.67 ± 25.14 ^#,$^	3.08 ± 0.39 ^#,$^

Cis, cisplatin (i.p. injection of 5 mg/kg in 0.9% saline) group; Cis + ADSCs, cisplatin group receiving ADSCs (one million cells, intravenous in tail vein); Cis + Rapa-ADSCs, cisplatin group receiving same number of ADSCs preconditioned with rapamycin (50 nM/L, 2 h prior to administration); * vs. control group (*p* < 0.01); ^#^ vs. Cis group (*p* < 0.01); ^$^ vs. Cis+ADSCs group (*p* < 0.05).

**Table 3:** The effect of rapamycin-preconditioned ADSCs on liver antioxidative potential; MDA, GPX and SOD, after cisplatin (Cis)-induced liver damage.

Group	MDA (nmol/g)	GPX (U/g)	SOD (U/g)
Control	87.38 ± 17.86	120 ± 8.07	197.2 ± 10.87
Cis	194.3 ± 16.55 ^*^	46.48 ± 17.94 *	70.89 ± 15.34 *
Cis + ADSCs	126.86 ± 15.83 *^,#^	78.96 ± 16.76 *^,#^	120.15 ± 27.82 *^,#^
Cis + Rapa-ADSCs	99.7 ± 15.36 ^#,$^	104.4 ± 20.64 ^#^	175.48 ± 14.88 ^#,$^

Cis, cisplatin (i.p. injection of 5 mg/kg in 0.9% saline) group; Cis + ADSCs, cisplatin group receiving ADSCs (one million cells, intravenous in tail vein); Cis + Rapa-ADSCs, cisplatin group receiving same number of ADSCs preconditioned with rapamycin (50 nM/L, 2 h prior to administration); * vs. control group (*p* < 0.01); ^#^ vs. Cis group (*p* < 0.01); ^$^ vs. Cis+ADSCs group (*p* < 0.05).

## Data Availability

Data is contained within the article.
